# The Association of Body Composition and Musculoskeletal Characteristics with Police Recruit Performance: A Cross-Sectional Study

**DOI:** 10.3390/jfmk10020132

**Published:** 2025-04-15

**Authors:** Vanessa R. Sutton, Myles C. Murphy, Callum J. McCaskie, Paola T. Chivers, Nicolas H. Hart, Jodie L. Cochrane Wilkie, Garth Allen, Jack Dalla Via

**Affiliations:** 1 Nutrition and Health Innovation Research Institute, School of Medical and Health Sciences, Edith Cowan University, Joondalup, WA 6027, Australia; vsutton0@our.ecu.edu.au (V.R.S.); j.dallavia@ecu.edu.au (J.D.V.); 2 Institute for Health Research, The University of Notre Dame Australia, Fremantle, WA 6160, Australia; paola.chivers@health.wa.gov.au (P.T.C.); nicolas.hart@uts.edu.au (N.H.H.); jodie.cochrane.wilkie@scu.edu.au (J.L.C.W.); 3 Exercise Medicine Research Institute, School of Medical and Health Sciences, Edith Cowan University, Joondalup, WA 6027, Australia; cmccaski@our.ecu.edu.au; 4 Western Australian Bone Research Collaboration, Fremantle, WA 6160, Australia; 5 Human Performance Research Centre, INSIGHT Research Institute, Faculty of Health, University of Technology Sydney, Moore Park, NSW 2021, Australia; 6 Caring Futures Institute, College of Nursing and Health Science, Flinders University, Bedford Park, SA 5042, Australia; 7 Centre for Healthcare Transformation, Faculty of Health, Queensland University of Technology, Kelvin Grove, QLD 4059, Australia; 8 Physical Activity, Sport and Exercise Research Theme, Faculty of Health, Southern Cross University, Bilinga, QLD 4225, Australia; 9 WA Police Academy, Western Australia Police Force, Joondalup, WA 6027, Australia; 10 Institute for Physical Activity and Nutrition, School of Exercise and Nutrition Sciences, Deakin University, Geelong, VIC 3220, Australia

**Keywords:** tactical, law enforcement, readiness, DXA, PQCT

## Abstract

**Objective**: Exploring how body composition and musculoskeletal characteristics relate to physical performance may provide insights for optimising training outcomes. We explored if body composition and musculoskeletal characteristics were associated with tactical and cardiorespiratory performance. **Methods**: A cross-sectional study of police recruits within the Western Australia Police Force was performed. Total and regional body composition was assessed using Dual-energy X-ray Absorptiometry, with the tibial morphology and mid-thigh muscle cross-sectional area assessed using peripheral Quantitative Computed Tomography. Tactical performance was measured with a Physical Performance Evaluation, and cardiorespiratory fitness assessed using the Beep Test. Variables that were significant in univariate regressions progressed to generalised linear models, assessing relationships between measures and performance outcomes. **Results**: Twenty-seven recruits aged 21–51 years (40.7% female) participated. Better tactical performance was associated with lower body fat percentage (*p* < 0.001), lower body mass index (*p* < 0.001), higher appendicular muscle mass (*p* = 0.005), and a lower proximal (66%) tibia polar cross-section moment of inertia (*p* = 0.007). Better cardiorespiratory fitness was associated with lower body fat percentage (*p* = 0.004), higher appendicular lean mass (*p* = 0.006), a lower proximal (66%) tibia polar cross-section moment of inertia (*p* = 0.005), and a higher mid-thigh muscle cross-sectional area (*p* < 0.001). **Conclusions**: Various body composition and musculoskeletal characteristics are associated with tactical performance and cardiorespiratory fitness in WA police recruits. Lower body fat percentage and higher appendicular muscle mass were associated with both better cardiorespiratory fitness and tactical performance, highlighting the potential relevance of these characteristics in preparing police recruits for operational duties.

## 1. Introduction

Police forces have minimum health standards for entry, including physical and psychological standards that potential recruits need to meet prior to consideration for entry into the police force [1,2,3]. The Western Australia (WA) Police Force Academy’s entry process requires applicants to pass standardised testing that includes psychological, medical, and physical components [3]. WA Police Force physical entry test protocols require applicants to demonstrate minimal levels of physical readiness to meet the demands of the 28-week academy training program. Tests include the 20 m multistage fitness test (beep test) to evaluate cardiorespiratory fitness [4] and a battery of agility, strength, power, and isometric muscular strength tests. This approach thereby focuses on functional capacity, as opposed to body composition, with the only demographic entrance criterion being aged 18 years or over [5].

However, due to global challenges in recruitment, police forces across Australia have reduced the difficulty level of some entry tests to improve recruitment rates [6,7]. Importantly, this change has diversified the applicant pool and resulted in a police force more representative of the general population. However, there are unknown consequences, particularly in the ability of applicants to complete the physical demands of training and the impact on injury rates. For example, some police force jurisdictions’ entry standards include raising the upper age limit (or removing it entirely) [5,8], which is problematic when older age is associated with more injuries [9].

Despite having met physical and medical entry requirements, police recruits can experience injuries that leave them unable to train or having to drop out of the recruit program [9] The prevalence of time-loss injuries is known to be as high as one in five recruits during the recruit training program [9]. This injury prevalence is comparable to injury rates observed in other tactical populations, such as military recruits [10,11,12]. Thus, proactive measures to mitigate injury risks are needed [9,13].

The injury incidence rate in Australian Police Force recruits have been reported as two injuries per 1000 training days [9] The most common locations of injury are the knee, shoulder, and lower leg, with the most common tissue injuries reported as ligament or joint capsule, followed by muscle or tendon [9] Further, a study in police force recruits demonstrated that injuries prior to police academy training directly impact performance [14]. Specifically, previous injuries that resulted in persistent disability were associated with poorer functional fitness test performance [14].

Research in police recruits examining associations between body composition, bone health, and physical performance is limited [15], as reduced performance during training has been associated with prior injury [14]. Understanding whether body composition and bone health are related to performance may help identify physical performance factors relevant to injury risk. Some research in military populations (who undergo comparable physical training) has examined body composition in relation to physical performance; however, these studies have tended to focus on strength-based tasks aligned with military demands rather than functional or cardiorespiratory performance relevant to police training [16,17].

To our knowledge, the only police-specific research examining the association of body composition and cardiorespiratory performance is in U.S. law enforcement recruits. This study included consumer-grade bioelectrical impedance measurements collected during routine practices by the police agency [18]. Greater skeletal muscle mass and lower fat mass were associated with better performance in isolated fitness tests, including muscular endurance, cardiorespiratory fitness, and agility [18]. However, the size of these associations were small. Further, the use of consumer-grade bioelectrical impedance, which has recognised limitations for estimating body composition and lacks specific validation for police populations, limits the accuracy of body composition estimates [19,20]. In addition, the isolated physical tests assess individual physical capacities separately rather than integrating multiple demands under occupationally relevant conditions as required in policing [18]. Although BMI and body mass are often reported in police recruit studies, with recruits typically falling within healthy or overweight ranges, the detailed examination of body composition in relation to performance is limited [1,21,22]. Bone characteristics have not been examined in police recruits, and studies exploring links between bone and physical performance are lacking in both police and military populations [15].

To address this gap, this exploratory study aimed to examine how baseline body composition and musculoskeletal characteristics, assessed using imaging-based methods, were associated with cardiorespiratory and tactical performance. We hypothesised that aspects of body composition, particularly greater lean mass and lower body fat percentage, would be associated with better performance across both cardiorespiratory fitness and functional performance outcomes.

## 2. Methods

### 2.1. Study Design

We performed a cross-sectional study of WA Police Force recruits who commenced recruit training at the WA Police Force Academy between January 2022 and October 2022.

### 2.2. Setting

All WA Police Force recruits are required to undergo a 28-week training program at the WA Police Force Academy [5] prior to becoming operational police force officers.

### 2.3. Participants

All participants in this study were WA Police Force recruits without medical conditions preventing physical training. However, all testing was performed prior to the recruits commencing formal academy training. All recruits were from WA, and none came from the International Transitional Officer program. The International Transitional Officer program is distinct from general recruit entry, as it only includes trained police officers from other countries and jurisdictions, requiring an abbreviated training program to bridge differences between forces. Other than being 18 years or older, no other demographic exclusion criteria were employed on prospective recruits.

### 2.4. Ethical Approvals

All participants in this study were provided with an electronic participation information letter and provided written, electronic, and informed consent before participation. This study was approved by the Edith Cowan University Human Research Ethics Committee (ID: 2020-01861-HART).

### 2.5. Data Collection

#### 2.5.1. Demographic Data

Self-reported demographic data were collected via Qualtrics using self-report surveys distributed to participants via email, which were submitted within the two weeks prior to commencing police force academy recruit training. A disability composite score was calculated based on self-reported pre-academy injuries. The disability composite score was based on self-reported responses to the neck disability index [23], Oswestry disability index [24], disabilities of the arm, shoulder, and hand score [25], Copenhagen hip and groin outcome score [26], knee injury and osteoarthritis outcome score [27], and foot and ankle outcome score [28].

Physical activity was assessed [29] utilising the bone physical activity questionnaire, in which recruits self-reported bone relevant load-bearing physical activity history [29]. This included the number of physical activities performed before and after age 18, the number of different training types, and the total number of training sessions per week in the past 12 months [29].

Radiological imaging, height, and weight were performed at the Edith Cowan University High-Performance Centre in Lathlain, Western Australia, the week before the recruits commenced police academy training. All recruits were instructed to wear comfortable clothing, such as sportswear, with all metallic items and jewellery removed and no shoes worn. Height was recorded to the nearest 0.1 cm (cm) using a stadiometer (Model 217, Seca, Hamburg, Germany). Weight was measured to the nearest 0.1 kg (kg) using electronic scales (Model 22089, Seca, Hamburg, Germany). Body mass index was calculated using these measurements in kg/m^2^.

#### 2.5.2. Cardiorespiratory and Tactical Performance

Physical performance data were collected following the WA Police Force Standard Operating Procedures by a Police Force Physical Training Instructor.

#### 2.5.3. Radiological Measures

Body composition was assessed using a total body Dual-energy X-ray Absorptiometry (DXA) scan (Hologic Horizon-A, Danbury, CT, USA). DXA operation was conducted by two qualified technicians (CJM and VRS), and a subsequent analysis of scans was conducted (CJM and JDV) on-site. Daily calibration routines were performed in accordance with the manufacturer specifications. The coefficient of variation (CV) for whole-body DXA scans in our facility, used by the same operator (CJM) for repeat scans on a subset of 30 individuals (males and females of varying ages and sizes), were as follows: total mass = 0.22%; LSTM = 0.41%; FM = 1.61% for the whole body [30,31]. Although hydration was not standardised, scan timing was controlled to minimise potential variability due to recent fluid or food intake [32].

Peripheral Quantitative Computed Tomography (pQCT; XCT-3000, Stratec Medizintechnik, Pforzeim, Germany) was used to scan cross-sections of the distal (4%) and proximal (66%) tibia and the mid (50%) femur. Tibial length (to the nearest 0.1 cm) was measured using constant-tension retractable measuring tape (Model 4414; Tech-Med Service, New York, NY, USA) from the bottom of the medial malleolus at the distal end of the tibia to the top of the tibial plateau at the knee joint. For both the tibia and femur scans, a scout view was performed to determine the distal end of each bone. The tibial length was then used to determine the 4% and 66% tibia sites and the 50% femur site. pQCT images were analysed in the Fiji image analysis platform [33] using the BoneJ plugin [34] as previously reported [35]. The distal tibia (4%) total bone area was analysed based on thresholds at 169 mg/cm^3^. The inner 45% of this was considered to be trabecular bone, determined by peeling single layers of pixels until 55% of the total bone area had been peeled away. For the 66% tibia, thresholds of 280 mg/cm^3^ and 710 mg/cm^3^ were used to determine the periosteal surface and cortical bone, respectively. Prior to analysis, all scans were assessed according to the visual inspection rating scale of participant movement and excluded where necessary [36]. The coefficient of variation for repeat tibial pQCT scans of the left lower leg on a subset of four individuals [35] by the same operator (CM) were as follows: tibial mass = 0.62%; tibial cross-sectional area = 0.80%; volumetric bone mineral density = 0.33%. A quality control cone phantom was also scanned every three days, and the coefficient of variation for total attenuation for repeat scans was 0.14% [30,31].

#### 2.5.4. Performance and Fitness Measures

The physical performance evaluation (in seconds) was used to capture tactical performance. While it is not presently validated, the WA Police Force has utilised this test as a police-specific functional task and for physical entrance standards for more than ten years [3]. In the physical performance evaluation, recruits sprint one lap of a 165 m running track without obstacles before progressing to a second lap with obstacles [3]. The obstacles include traversing a 6 m balance beam, clearing a 1.8 m fence, vaulting a 1.2 m brick wall, jumping a 1.5 m length sand-pit, hurdling over two 60 cm hurdles with a 5 m distance between them, a 15 m farmers carry of two 20 kg dumbbells, climbing through open windows at 1.2 and 1.5 m height, scaling and clearing a 2.8 m high cyclone fence, and picking up and carrying a 40 kg sandbag for 15 m [3]. The physical performance evaluation is a timed test, with a faster completion time indicative of better work-specific functional capacity.

The beep test is a validated 20-metre shuttle run test that assesses cardiorespiratory fitness (estimating VO^2^ max using a running task) [37]. Each squad performed the beep test together. Recruits are then assigned a cut-off level (e.g., Level 8), with a higher level representing better cardiorespiratory fitness [37].

### 2.6. Variables

#### 2.6.1. Demographic Variables

The following demographic variables were included: age (years calculated from date of birth); sex (male, female); height (cm); weight (kg); body mass index (kg/m^2^, calculated from height and weight); disability composite (percentage); a bone physical activity questionnaire (current score, previous score, total score).

#### 2.6.2. Performance and Fitness Variables

The following tactical performance and cardiorespiratory fitness results were included: timed results (seconds) for the physical performance examination; highest level (e.g., level 8) successfully completed in the beep test.

#### 2.6.3. Radiological Measures

The following variables were quantified using DXA: body fat percentage (%); waist circumference (cm); appendicular lean mass (kg); hip areal bone mineral density (g/cm^2^); femoral neck areal bone mineral density (g/cm^2^); lumbar spine areal bone mineral density (g/cm^2^).

The following variables were quantified using pQCT at the distal 4% tibia: trabecular volumetric bone mineral density (mg/cm^3^); bone strength index (g/cm^4^). The following variables were quantified using pQCT at proximal 66% tibia: cortical bone area (mm^2^); polar cross-sectional moment of inertia (mg/cm); tibia cortical volumetric bone mineral density (mg/cm^3^) [38]. The following variables were quantified using pQCT at the proximal 50% femur: mid-thigh muscle cross-sectional area (MuA; cm^2^) [38].

The selected DXA and pQCT radiological variables were chosen for their direct relevance to performance metrics in a tactical population while minimising redundancy. Body fat percentage and waist circumference are known to be associated with impaired physical performance [39,40]. Appendicular lean mass is the accumulation of lean mass summated across all areas of the appendicular regions (i.e., upper limbs and lower limbs) and is relevant for physical tasks [41]. Tibia volumetric bone mineral density is associated with a history of impact exercise, which is important given the physical demands of tasks encountered in police force employment [42]. At the 4% tibia, trabecular volumetric bone mineral density and bone strength index were selected for investigation due to their orientation along stress-adaptive pathways, making them sensitive to impact forces [43]. At the 66% tibia, cortical volumetric bone mineral density, cortical area, and polar cross-sectional moment of inertia were selected for investigation due to their indication of bone rigidity and resistance to bending or torsional forces, which are important in load-bearing tasks [44]. Muscle cross-sectional area was analysed at the femur, where it most accurately indicates lower-limb power [45,46].

#### 2.6.4. Consideration of Other Variables

This exploratory study focused on the relationship between body composition, musculoskeletal characteristics, and performance outcomes. While we recognise that a range of other factors including, but not limited to, nutrition, sleep, menstrual cycles, and smoking can influence performance, examining these were beyond the intended scope of the present analysis. 

### 2.7. Data Management

Data linkage was performed to merge demographic, body composition, musculoskeletal, and performance data. Data were securely transferred electronically to the research team to be linked to the participant’s data, which was then de-identified in a coded numerical format. The physical performance tests (i.e., beep test and physical performance evaluation) were conducted as routine practice on day one of the recruit training to ascertain baseline measures.

### 2.8. Data Analysis

IBM SPSS Statistics (v29.0.2.0) was used for all data analyses. All variables were described using mean, standard deviation, minimum–maximum, median, and 25th–75th inter-quartile range for the total sample and split by self-reported sex (male/female). Data were examined for normality via the Shapiro–Wilk test. Among the variables, physical performance evaluation completion time, age, bone physical activity questionnaire current score, and injury composite satisfied the assumption of normality. All other variables did not.

The exploratory analysis of these data was divided into two phases:

(i) Univariate linear regressions (or the Kruskal–Wallis H-test for non-parametric data) for the association of radiological measures to either cardiorespiratory fitness or tactical performance;

(ii) Multivariate generalised linear modelling exploring the association between radiological variables with cardiorespiratory performance or tactical performance, adjusted for covariates known to influence the radiological measure (e.g., age or sex as informed by the Supplementary Materials). Only radiological variables with a *p*-value of less than 0.1 from phase one were included in multivariable modelling. Where height and/or weight were significant, only BMI progressed to phase two analysis, given it is a construct of both. A stepwise approach was used for model fit, including demographic variables and interaction terms, when it improved overall model fit. The Akaike Information Criterion was used for model fit, with lower values indicating a better model of fit. Sex was included as a main effect, and interaction terms with sex were explored to assess whether associations varied between males and females. Stratified analyses were not conducted due to sample size constraints. All final model residuals were inspected, and no violations were noted. Statistical significance was accepted when *p* < 0.05 in phase two.

## 3. Results

Twenty-seven (*n* = 27) WA Police Force recruits were included in this study, with all characteristics, split by sex, presented in Table 1 and Table 2.

### 3.1. Participant Characteristics

#### 3.1.1. Demographics

There were 16 males (59.3%) and 11 females (40.7%). The female recruits were significantly older (*p* = 0.004) than the male recruits [female mean (SD) age = 35 (11) years; male mean (SD) age = 28 (7) years]. Participants had a mean (SD) bone physical activity questionnaire current score of 5.4 (3.7), a previous score of 24.3 (19.1), and a total score of 14.9 (10.1). The mean (SD) injury composite score based on responses to patient-reported outcome measure questionnaires was 5.8 (7.3).

**Table 1:** Participant characteristics.

	Males	Females	Combined
	M (SD)	M (SD)	M (SD)
n	16	11	27
Age (years)	28.2 (7.1)	34.6 (10.7)	30.8 (9.1)
Height (cm)	178.9 (7.3)	168.0 (4.6)	174.5 (8.3)
Weight (kg)	78.1 (10.9)	64.5 (9.1)	72.7 (12.0)
Body Mass Index (kg/cm^2^)	24.5 (3.7)	23.1 (3.9)	23.9 (3.7)
Physical Performance Examination (seconds)	109.7 (20.6)	167.8 (53.4)	133.4 (46.8)
Beep Test ^a^ (level)	9.3 (1.0)	7.5 (1.5)	8.5 (1.5)
BPAQ—Current Score	5.3 (3.2)	5.7 (4.5)	5.4 (3.7)
BPAQ—Previous Score	26.9 (21.6)	20.6 (14.7)	24.3 (19.1)
BPAQ—Total Score	16.1 (11.4)	13.1 (7.9)	14.9 (10.1)
Injury Composite ^b^	3.5 (4.5)	8.5 (9.3)	5.8 (7.3)

Legend: ^a^ n = 26 (males n = 15, females n = 11); ^b^ n = 22 (males n = 12, females n = 10); M = mean; SD = standard deviation; n = number; BPAQ = bone physical activity questionnaire.

#### 3.1.2. Cardiorespiratory Performance

Participants had a mean (SD) beep test score of 8.5 (1.5) levels.

#### 3.1.3. Tactical Performance

Participants had a mean (SD) physical performance evaluation completion time of 133.4 (46.8) seconds.

#### 3.1.4. Radiological Measures

Participants had a mean (SD) body fat of 21.8 (5.6) percent, waist circumference of 90.4 (10.4) cm, and appendicular lean mass of 25.6 (6.1) kg. All other radiological measures are reported in Table 2.

**Table 2:** Participant radiological profiles.

	Males	Females	Combined
	M (SD)	M (SD)	M (SD)
n	16	11	27
Body Fat (%)	18.9 (3.7)	25.9 (5.3)	21.8 (5.6)
Waist Circumference (cm)	93.7 (9.6)	85.8 (10.2)	90.4 (10.4)
Appendicular Lean Mass (kg)	29.2 (4.8)	20.2 (2.8)	25.6 (6.1)
Hip aBMD (g/cm^2^)	1.1 (0.2)	1.0 (0.1)	1.0 (0.2)
Femoral Neck aBMD (g/cm^2^)	1.0 (0.2)	0.9 (0.2)	1.0 (0.2)
Lumbar Spine aBMD (g/cm^2^)	1.1 (0.1)	1.1 (0.2)	1.1 (0.1)
Distal (4%) Tibia Trabecular vBMD (mg/cm^3^)	272.8 (30.9)	229.1 (33.8)	255.0 (38.3)
Distal (4%) Tibia Bone Strength Index (g/cm^4^)	1.6 (0.4)	0.9 (0.3)	1.3 (0.4)
Proximal (66%) Tibia Cortical vBMD (mg/cm^3^)	1108.3 (22.5)	1113.0 (25.4)	1110.2 (23.3)
Proximal (66%) Tibia Cortical Area (mm^2^)	484.9 (75.1)	374.6 (48.2)	440.0 (84.8)
Proximal (66%) Tibia Polar Cross-Sectional Moment of Inertia (mg/cm)	8390.9 (1922.3)	4974.7 (1120.4)	6999.1 (2353.9)
Mid-thigh Muscle Cross-Sectional Area ^a^ (cm^2^)	158.4 (25.5)	118.7 (21.2)	142.8 (30.6)

Legend: ^a^ n = 23 (males n = 14, females n = 9). M = mean; SD = standard deviation; n = number; aBMD = areal bone mineral density; vBMD = volumetric bone mineral density.

### 3.2. Association Between DXA-Derived Radiological Measures and Tactical Performance

Univariate models were performed on each DXA-derived measure and are included in the Supplementary Material.

Hip areal bone mineral density, femoral neck areal bone mineral density, lumbar spine areal bone mineral density, and waist circumference were not associated with tactical performance. The following models are only inclusive of the radiological measurements that proceeded to phase two of statistical analysis.

Body Fat Percentage (Model 1). A higher body fat percentage was associated with a slower physical performance evaluation completion time (β = 8.374, *p* < 0.001; Table 3). A significant age and body fat percentage interaction was detected with older recruits (aged over 30 years), with higher body fat percentage associated with even slower physical performance evaluation completion times (β = −4.915, *p* = 0.008; Table 3).

Body Mass Index (Model 2). A higher body mass index was associated with a slower physical performance evaluation completion time (β = 10.759, *p* < 0.001; Table 3). A significant sex and body mass index interaction was detected with the female recruits’ performance evaluation completion time slower with higher BMI (β = −9.893, *p* < 0.001; Table 3).

Appendicular Lean Mass (Model 3). A higher appendicular lean mass was associated with a faster performance evaluation completion time (β = −3.151, *p* = 0.005; Table 3).

### 3.3. Association Between pQCT-Derived Radiological Measures and Tactical Performance

Univariate models were performed on each pQCT-derived measure and are included in the Supplementary Materials. Trabecular volumetric bone mineral density and bone strength index at the distal 4% tibia, cortical bone area, cortical volumetric bone mineral density at the proximal 66% tibia, and muscle cross-sectional area at the 50% femur did not progress to phase two analysis. The following model is only inclusive of the radiological measurement that proceeded to phase two of statistical analysis.

Polar Cross-Section Moment of Inertia—66% site (Model 1). A higher polar cross-section moment of inertia at the 66% site was associated with a slower PPE completion time (β = 0.024, *p* = 0.007; Table 4). Female physical performance completion time varied more in relation to polar cross-sectional moment of inertia than in males (significant sex and polar interaction). For example, female recruits with a comparably high polar cross-sectional moment of inertia demonstrated slower completion times than male recruits (β = −0.025, *p =* 0.012; Table 4).

### 3.4. Association Between DXA-Derived Radiological Measures and Cardiorespiratory Performance

Univariate models were performed on each DXA-derived measure and are included in the Supplementary Materials. Hip areal bone mineral density, femoral neck areal bone mineral density, lumbar spine areal bone mineral density, and waist circumference did not progress to phase two analysis. The following models are only inclusive of the radiological measurements that proceeded to phase two of statistical analysis.

Body Fat Percentage (Model 1). A higher body fat percentage was associated with lower cardiorespiratory fitness (β = −0.133, *p* = 0.004; Table 5).

Body Mass Index (Model 2). Body mass index showed no association with cardiorespiratory fitness (β = −0.061, *p* = 0.322; Table 5).

Appendicular Lean Mass (Model 3). A higher appendicular lean mass was associated with higher cardiorespiratory fitness (β = −0.065, *p* = 0.006; Table 5).

### 3.5. Association Between pQCT-Derived Radiological Measures and Cardiorespiratory Performance

Univariate models were performed and are included in the Supplementary Materials. Trabecular volumetric bone mineral density and bone strength index at the distal 4% tibia did not progress to phase two analysis. The following models are only inclusive of the radiological measurements that proceeded to phase two of statistical analysis.

Cortical Volumetric Bone Mineral Density 66% Tibia (Model 1). Cortical volumetric bone mineral density showed no association with cardiorespiratory fitness (β = −0.016, *p* = 0.093; Table 6).

Cortical Area—Proximal 66% Tibia (Model 2). Cortical area showed no association with cardiorespiratory fitness (β = 0.001, *p* = 0.836; Table 6).

Polar Cross-Sectional Moment of Inertia—Proximal 66% Tibia (Model 3). A higher polar cross-sectional moment of inertia was significantly associated with better cardiorespiratory fitness (β = 0.000, *p* = 0.005; Table 6).

Muscle Area (Cross-sectional)—Mid-Thigh (Model 4). A higher mid-thigh muscle cross-sectional area was associated with better cardiorespiratory fitness (β = 0.026, *p* < 0.001; Table 6).

## 4. Discussion

Our study explored associations between demographic, body composition, and musculoskeletal variables with tactical performance and cardiorespiratory fitness in police force recruits. We identified that DXA-derived measures displayed comparable associations with tactical performance and cardiorespiratory fitness, whereas significant pQCT-derived variables were different for tactical performance and cardiorespiratory fitness.

Body fat percentage, body mass index, appendicular lean mass, and polar cross-sectional moment of inertia (66% tibia) were associated with tactical performance. Conversely, hip areal bone mineral density, femoral neck areal bone mineral density, lumbar spine areal bone mineral density, waist circumference, trabecular volumetric bone mineral density (4% tibia), bone strength index (4% tibia), cortical bone area (66% tibia), cortical volumetric bone mineral density (66% tibia), and muscle cross-sectional area (50% femur) were not associated with tactical performance.

Body fat percentage, appendicular lean mass, polar cross-sectional moment of inertia (66% tibia), and muscle cross-sectional area (50% femur) were associated with cardiorespiratory performance. Conversely, hip areal bone mineral density, femoral neck areal bone mineral density, lumbar spine areal bone mineral density, body mass index, waist circumference, trabecular volumetric bone mineral density (4% tibia), bone strength index (4% tibia), cortical bone area (66% tibia), and cortical volumetric bone mineral density (66% tibia) were not associated with cardiorespiratory fitness.

Our research aligns with previous studies demonstrating that excess body fat can impair mobility and endurance, which are critical for completing physically demanding tasks [47,48,49]. However, while previous research in some military populations has suggested that body mass index does not impair performance [16], our findings indicate the possibility that higher body mass index is linked to worse tactical performance in police recruits. The contrast in findings may reflect differences in body composition between cohorts at a given BMI. Further, our results suggest these differences may be due to a greater proportion of fat mass in police recruits rather than lean mass, as lean mass in our cohort was associated with better tactical performance. In addition, differences in the physical tasks used to assess tactical performance between military and police recruits may also contribute to variations in BMI-performance associations [50]. The negative impact of body fat percentage on police specific tactical performance, emphasises the importance of managing overall body fat and fat distribution in recruits. In contrast, recruits with higher appendicular lean mass have better tactical performance, accentuating the role of muscle mass in supporting strength and agility during physically demanding tasks. This finding is consistent with the existing literature linking lean mass to improved functional outcomes in tactical and physically active populations [51,52].

Higher polar cross-sectional moment of inertia values (proximal 66% tibia) were associated with slower physical performance evaluation completion times (particularly in female recruits) and lower cardiorespiratory fitness. While this was unexpected, our study design does not allow us to determine whether this association reflects body size, long-term loading history, or other morphological factors [46]. Given the small sample and the absence of detailed limb dimension or training history data, these results should be interpreted cautiously and warrant further investigation in larger, more stratified samples. Values of polar cross-sectional moment of inertia were reported as they represent the bone’s ability to resist bending forces, which is important for physical demands and load-bearing tasks [46], inherent to police recruit training and operational duties. While these bone measures provide important insights into bone health, their relationship with functional performance is not well understood. One possible explanation for better bone strength being associated with poorer functional performance is the influence of body weight. Bone is known to adapt to mechanical loading, particularly in weight-bearing bones such as the tibia [53]. Therefore, increased loading during daily activities due to higher body weight has been suggested to contribute to improved bone strength over a prolonged period [54]. It is important to note that though previous research has linked higher body mass to improved bone health, there is also a potential link to increased fracture risk alongside benefits to bone modelling in this population [54]. Consistent evidence shows that being overweight and being obese (e.g., higher body fat percentage) is associated with greater bone density [54]. This explanation is supported by our observation that higher body fat percentage and body mass index were also associated with poorer tactical performance, likely due to excess weight impairing agility and speed with testing. However, further research is necessary to explore the interaction between body fat, bone and functional performance in tactical populations, especially given that some other DXA and pQCT bone measures were not associated with functional performance in phase one of our analysis.

Our findings demonstrated that higher body fat percentage was negatively associated with cardiorespiratory fitness, indicating a potential link between excess body fat and reduced cardiorespiratory fitness in police recruits. These results align with previous research suggesting that elevated body fat may be associated with lower aerobic capacity [40,55], which can affect performance in endurance-based tasks like the beep test. In contrast, higher appendicular lean mass was positively associated with beep test performance, suggesting that muscle mass may play a role in aerobic capacity [56]. Optimising body composition, particularly by increasing lean mass and reducing body fat may, therefore, supports improved cardiorespiratory fitness and endurance performance in tactical populations.

Although it is unlikely that improved bone health is directly responsible for improved fitness, it is possible that it is associated with reduced injury risk during activities performed by police officers. For example, military recruits with more favourable tibial bone structure and strength are shown to have reduced risk of bone stress injuries during training [57]. However, future research is needed to explore the role of specific bone characteristics in predicting injury risk and performance in other tactical populations with a lower incidence of bone stress injury [9].

In addition to our findings, the use of well-validated methods and equipment [58] in this study has provided evidence of the significant relationship between body composition and musculoskeletal health of contemporary police recruits. This emphasises the benefit of data-driven, multi-modality [58] approaches to assessing body composition and physical performance in recruits. We identified that key factors such as body fat percentage and lean mass could help refine pre-training assessments, allowing police academies to better identify recruits who may benefit from tailored fitness programs to improve outcomes and potentially reduce injury risk. However, in previous qualitative work, police recruits indicated that they were opposed to measures of body fat percentage being introduced and preferred the focus is on performance, so the implementation of this may be problematic [59].

Advanced imaging technologies like DXA and pQCT offer reliable assessments of body composition and bone characteristics [58], however, their high cost and complexity limit practical application in routine assessments [20]. If available, these imaging methods could be useful in pre-screening recruits to predict performance and potentially improve training individualisation. However, for broader, real-world use, practical and cost-effective tools such as direct segmental multifrequency bioelectrical impedance analysis scales for body fat percentage may offer more accessible options for ongoing evaluation [19,20]. While previous studies have used consumer-grade BIA, these devices differ substantially from direct segmental multifrequency models in both accuracy and applicability [19,20]. It is important to note that bioelectrical impedance analysis scale accuracy may vary in those with an obese range body mass index or those who are not euhydrated [60], however, whilst not as accurate as DXA [20], there may be benefits in direct segmental multifrequency bioelectrical impedance analysis use in the police recruit screening setting due to portability, cost, and comparable efficacy.

Future research should focus on validating easy-to-use, commercially available tools that can evaluate body fat percentage in police force recruits to determine if they can serve as reliable proxies for imaging-based measures and whether they are similarly associated with performance and fitness outcomes. If these tools can reliably estimate body composition in relation to suboptimal performance, similar to our methods and consequent findings, they could facilitate the routine monitoring of recruits. Further, they may inform the development of individually tailored training for recruits with suboptimal performance and those at risk of injury. Ultimately, by integrating both advanced and accessible assessment methods, police academies could optimise training programs, improve recruit performance, and potentially further reduce injury risks, leading to enhanced readiness and resilience not only for police recruits but also for other tactical populations.

## 5. Limitations

The cross-sectional nature of our study meant that we were unable to explore causal inferences about relationships of body composition and bone characteristics with performance. A future study using longitudinal design would allow for the exploration of changes in body composition and bone characteristics over time on performance. Males and females have different bone health outcomes during growth and development, including different propensities for skeletal robustness and strength due to various hormonal and other factors that also apply in adulthood and beyond. Thus, combining sexes in research may be problematic, despite our approach to assess sex as a main effect and interaction in statistical modelling [61,62]. We also had a relatively small sample size, with 27 participants. This limits the generalisability of our findings to the broader police recruit population and may reduce the statistical power to detect more subtle associations between body composition, bone characteristics, and performance outcomes.

While the tactical performance outcome used in this study was the embedded testing adopted by WA police, it has not been externally validated. To our knowledge, no comparable police obstacle test has been successfully externally validated. However, this study did not aim to validate the performance measure, and it was selected as it reflects the standard assessment used operationally. Future longitudinal studies could help to clarify the temporal direction of these associations.

Although we explored interaction effects and identified several statistically significant subgroup associations (e.g., sex and age interactions), these findings are exploratory and may be sensitive to sample variability. Replication in larger samples is needed to reduce uncertainty and to support a more robust interpretation of these effects. Participation was voluntary, and as with any observational study, the potential for self-selection bias must be acknowledged.

## 6. Conclusions

This study has demonstrated associations between body composition, bone characteristics, and police-specific physical performance testing in police recruits. Factors such as body fat percentage, appendicular lean mass, and bone strength (measured using Ipolar) are associated with tactical and cardiorespiratory performance. These findings provide a preliminary foundation for future longitudinal research into the role of body composition and musculoskeletal health in police recruit performance and may inform future strategies to support performance and reduce injury risk.

## Figures and Tables

**Table 3:** Association between DXA-derived radiological measures and tactical performance.

Model	Variable	AIC	β	95% CI		SE	*p*-Value
				Lower	Upper		
Model 1	Intercept	258.32	−47.293	−104.269	9.684	29.070	0.104
	Body Fat Percentage		8.374	6.084	10.664	1.168	**<0.001**
	Age (29 yrs. and below)		96.484	15.06	177.908	41.544	**0.02**
	Interaction of Age ≥ 30 and Body Fat Percentage		−4.915	−8.555	−1.275	1.857	**0.008**
Model 2	Intercept	261.47	−80.450	−176.179	15.280	48.842	0.100
	Body Mass Index		10.759	6.662	14.857	2.091	**<0.001**
	Sex (male)		168.963	39.891	298.034	65.854	**0.010**
	Interaction of Sex and Body Mass Index		−9.893	−15.284	−4.502	2.751	**<0.001**
Model 3	Intercept	276.12	138.31	61.972	214.649	38.949	**<0.001**
	Age		2.456	0.991	3.922	0.748	**0.001**
	Appendicular Lean Mass		−3.151	−5.361	−0.941	1.128	**0.005**

Legend: β = Beta estimate, % = percentage, CI = confidence interval, SE = standard error. Bolded *p*-values represent significance at <0.05.

**Table 4:** Association between pQCT-derived radiological measures and tactical performance.

Model	Variable	AIC	β	95% CI		SE	*p*-Value
				Lower	Upper		
Model 1	Intercept	273.50	46.34	−43.286	135.966	45.728	0.311
	Proximal (66%) Tibia Polar Cross-Section Moment of Inertia (mg/cm^2^)		0.024	0.007	0.042	0.009	**0.007**
	Sex (Male)		68.738	−46.254	183.731	58.671	0.241
	Interaction of Polar Cross-Section Moment of Inertia and Sex		−0.025	−0.045	−0.006	0.010	**0.012**

Legend: β = Beta estimate, % = percentage, CI = confidence interval, SE = standard error. Bolded *p*-values represent significance at <0.05.

**Table 5:** Association between DXA-derived radiological measures and cardiorespiratory performance.

Model	Variable	AIC	β	95% CI		SE	*p*-Value
				Lower	Upper		
Model 1	Intercept	82.70	10.907	8.490	13.325	1.233	**<0.001**
	Sex (Male)		0.897	−0.115	1.909	0.516	0.082
	Body fat percentage		−0.133	−0.224	−0.043	0.046	**0.004**
Model 2	Intercept	89.02	8.855	5.953	11.756	1.480	**<0.001**
	Sex (Male)		1.923	1.007	2.838	0.467	**<0.001**
	Body mass index		−0.061	−0.183	0.060	0.064	0.331
Model 3	Intercept	86.39	7.376	4.937	9.816	1.245	**<0.001**
	Age		0.123	0.053	0.193	0.036	**<0.001**
	Appendicular lean mass		−0.065	−0.112	−0.019	0.024	**0.006**

Legend: β = Beta estimate, % = percentage, CI = confidence interval, SE = standard error, DXA = Dual-energy X-ray Absorptiometry. Bolded *p*-values represent significance at <0.05.

**Table 6:** Association between pQCT-derived radiological measures and cardiorespiratory fitness.

Model	Variable		β	95% CI		SE	*p*-Value
				Lower	Upper		
Model 1	Intercept	87.27	25.694	4.404	46.984	10.862	**0.018**
	Sex (Male)		1.715	0.841	2.589	0.446	**<0.001**
	Proximal (66%) Cortical Volumetric Bone Mineral Density		−0.016	−0.036	0.003	0.010	0.093
Model 2	Intercept	89.91	7.743	4.936	10.549	1.432	**<0.001**
	Sex (Male)		1.915	0.633	3.155	0.633	**0.002**
	Proximal (66%) Tibia Cortical Area (mm^2^)		−0.001	−0.008	0.006	0.004	0.836
Model 3	Intercept	89.40	8.811	6.710	10.912	1.072	**<0.001**
	Age		−0.071	−0.120	−0.022	0.025	**0.004**
	Proximal (66%) Tibia Polar Cross-Sectional Moment of Inertia (mg/cm)		0.000	8.108^−5^	0.000	9.690^−5^	**0.005**
Model 4	Intercept	72.63	6.826	4.216	9.435	1.332	**<0.001**
	Age		−0.063	−0.114	−0.013	0.026	**0.014**
	Mid-thigh Muscle Cross-Sectional Area (cm^2^)		0.026	0.012	0.041	0.007	**<0.001**

Legend: β = Beta estimate, % = percentage, CI = confidence interval, SE = standard error. Bolded *p*-values represent significance at <0.05.

## Data Availability

The data for this research are not available for sharing, but they may be provided upon request if approved by the Western Australia Police Force.

## Supplementary Materials

The following supporting information can be downloaded at: https://www.mdpi.com/article/10.3390/jfmk10020132/s1.
