# Supplementary material for: The Association of Body Composition and Musculoskeletal Characteristics with Police Recruit Performance: A Cross-Sectional Study
Source: J Funct Morphol Kinesiol. 2025 Apr 15;10(2):132. doi: 10.3390/jfmk10020132 (PMC12015768; doi:10.3390/jfmk10020132)
Supplement: Supplementary file 1 [file jfmk-10-00132-s001.zip › jfmk-3498199-supplementary.pdf]

Section S1. Association between age and sex with study variables

Table S1. Association of sex with study variables

Dependent	Independent	n	Test Statistic	Significance
Sex (Male / Female)	Weight (kg)	27	9.060	***0.003***
	Height (cm)	27	12.110	***<0.001***
	Body Mass Index (kg/cm^2)	27	0.879	0.348
	Hip Bone Mineral Density (g/cm^2)	27	3.516	0.061
	Femoral Neck Bone Mineral Density (g/cm^2)	27	2.340	0.126
	Spine Bone Mineral Density (g/cm^2)	27	0.197	0.657
	Waist Circumference (cm)	27	3.897	***0.048***
	Body Fat Percentage (%)	27	10.930	***<0.001***
	Appendicular Lean Mass (kg)	27	16.370	***<0.001***
	Beep Test (level)	26	8.951	***0.003***
	Physical Performance Examination (seconds)	26	17.020	***<0.001***
	Distal 4% Tibia, volumetric Bone Mineral Density (mg/cm^3)	27	9.665	***0.002***
	Distal 4% Tibia, Bone Strength Index (g/cm^4)	27	13.697	***<0.001***
	Proximal 66% Tibia, Cortical Bone Mineral Density (mg/cm^3)	27	0.412	0.521
	Proximal 66% Tibia, Cortical area (mm^2)	27	12.623	***<0.001***
	Proximal 66% Tibia, Polar Cross-Sectional Moment of Inertia (mg/cm)	27	15.584	***<0.001***
	Mid-thigh, Muscle Cross-Sectional Area (cm^2)	23	9.921	***0.002***

Table S2. Association of sex with variables

Dependent	Independent	n	Test Statistic	Significance
Age (Years)	Weight (kg)	26	0.125	0.435
	Height (cm)	26	-35.127	0.104
	Body Mass Index (kg/cm^2)	26	0.819	***0.083***
	Hip Bone Mineral Density (g/cm^2)	26	-12.294	0.281
	Femoral Neck Bone Mineral Density (g/cm^2)	26	-9.537	0.347
	Spine Bone Mineral Density (g/cm^2)	26	-7.371	0.593
	Waist Circumference (cm)	26	0.238	0.170
	Body Fat Percentage (%)	26	0.727	***0.021***
	Appendicular Lean Mass (kg)	26	-0.127	0.676
	Beep Test (level)	25	-2.749	***0.022***
	Physical Performance Examination (seconds)	26	0.100	***0.006***
	Distal 4% Tibia, volumetric Bone Mineral Density (mg/cm^3)	27	16.810	0.398
	Distal 4% Tibia, Bone Strength Index (g/cm^4)	27	15.479	0.490
	Proximal 66% Tibia, Cortical Bone Mineral Density (mg/cm^3)	27	18.653	0.287
	Proximal 66% Tibia, Cortical area (mm^2)	27	13.272	0.653
	Proximal 66% Tibia, Polar Cross-Sectional Moment of Inertia (mg/cm)	27	11.984	0.745
	Mid-thigh, Muscle Cross-Sectional Area (cm^2)	23	14.565	0.483

Section S2. Association between DXA-derived radiological measurements and tactical performance

Dependent	Independent	n	β	CI Lower	CI Upper	ANOVA F	Significance
Physical Performance Evaluation (Tactical Performance)	Body Mass Index (kg/cm^2)	26	3.405	-1.484	8.295	2.057	0.164
	Waist Circumference (cm)	26	0.427	-1.413	2.267	0.229	0.637
	Body Fat Percentage (%)	26	6.756	4.688	8.824	45.269	***<0.001***
	Appendicular Lean Mass (kg)	26	-3.463	-6.308	-0.618	6.284	***0.019***
	Hip Bone Mineral Density (g/cm^2)	26	-28.162	-148.173	91.849	0.234	0.633
	Femoral Neck Bone Mineral Density (g/cm^2)	26	-2.770	-109.632	104.093	0.003	0.958
	Spine Bone Mineral Density (g/cm^2)	26	-14.197	-158.487	130.094	0.041	0.841

Section S3. Association between DXA-derived radiological measurements and cardiovascular fitness

Dependent	Independent	n	β	CI Lower	CI Upper	ANOVA F	Significance
Beep Test (Cardiovascular Fitness)	Body Mass Index (kg/cm^2)	25	-0.005	-0.171	0.162	0.003	0.956
	Waist Circumference (cm)	25	0.140	-0.046	0.074	0.243	0.626
	Body Fat Percentage (%)	25	-0.183	-0.265	-0.101	21.296	***<0.001***
	Appendicular Lean Mass (kg)	25	0.132	0.046	0.291	9.961	***0.004***
	Hip Bone Mineral Density (g/cm^2)	26	1.021	-2.867	4.908	0.294	0.593
	Femoral Neck Bone Mineral Density (g/cm^2)	26	0.296	-3.158	3.750	0.031	0.861
	Spine Bone Mineral Density (g/cm^2)	25	1.075	-3.568	5.718	0.228	0.637

Section S4. Association between pQCT-derived radiological measurements and tactical performance

Dependent	Independent	n	β	CI Lower	CI Upper	ANOVA F	Significance
Physical Performance Evaluation (Tactical Performance)	Distal 4% Tibia, volumetric Bone Mineral Density (mg/cm^3)	26	-0.087	-0.589	0.414	0.128	0.723
	Distal 4% Tibia, Bone Strength Index (g/cm^4)	26	-24.889	-67.310	17.532	1.460	0.238
	Proximal 66% Tibia, Cortical Bone Mineral Density (mg/cm^3)	26	0.229	-0.591	1.050	0.332	0.570
	Proximal 66% Tibia, Cortical area (mm^2)	26	-0.151	-0.370	0.067	2.034	0.166
	Proximal 66% Tibia, Polar Cross-Sectional Moment of Inertia (mg/cm)	26	-0.007	-0.015	0.001	3.660	***0.067***
	Mid-Thigh, Muscle Cross-Sectional Area (cm^2)	22	-0.408	-1.119	0.304	1.420	0.247

Section S5. Association between pQCT-derived radiological measurements and cardiovascular fitness

Dependent	Independent	n	β	CI Lower	CI Upper	ANOVA F	Significance
Beep Test (Cardiovascular Fitness)	Distal 4% Tibia, volumetric Bone Mineral Density (mg/cm^3)	25	0.006	-0.010	0.022	0.681	0.417
	Distal 4% Tibia, Bone Strength Index (g/cm^4)	25	1.041	-0.305	2.386	2.547	0.124
	Proximal 66% Tibia, Cortical Bone Mineral Density (mg/cm^3)	25	-0.022	-0.048	0.004	3.032	***0.094***
	Proximal 66% Tibia, Cortical area (mm^2)	25	0.007	0.000	0.014	4.343	***0.048***
	Proximal 66% Tibia, Polar Cross-Sectional Moment of Inertia (mg/cm)	25	0.000	0.000	0.001	5.807	***0.024***
	Femur (Mid-Thigh), Muscle Cross-Sectional Area (cm^2)	21	0.026	0.008	0.044	9.282	***0.006***
